# Point-of-care Ultrasound Diagnosis of Acute Abdominal Aortic Occlusion

**DOI:** 10.5811/cpcem.2019.11.44311

**Published:** 2020-01-23

**Authors:** Benjamin Bloom, Ryan Gibbons, Dov Brandis, Thomas G. Costantino

**Affiliations:** * Temple University Hospital, Department of Emergency Medicine, Philadelphia, Pennsylvania; † Lewis Katz School of Medicine at Temple University, Department of Emergency Medicine, Philadelphia, Pennsylvania

## Abstract

Acute aortic occlusion is an emergent vascular condition not encountered routinely. Given its varied presentations, including neurovascular deficits and mimicking an acute abdomen, the diagnosis is often delayed causing increased morbidity and mortality. We present a case of acute abdominal aortic occlusion masquerading as sudden onset lower extremity pain and weakness in an 86-year-old female requiring emergent thrombectomy. This is only the second case report to discuss the use of point-of-care ultrasound to expedite diagnosis and management.^1^

## INTRODUCTION

Acute aortic occlusion (AAO) is a rare but potentially devastating vascular emergency. It can be secondary to an embolism, thrombus, or aneurysmal disease.^2^–^5^ Given its varied presentations, including neurovascular deficits, the diagnosis is often challenging and delayed, impeding time to revascularization and causing significant morbidity and mortality between 21–74%.^2^–^5^ Traditionally, computed tomography (CT) angiography has been the diagnostic modality of choice. However, issues including patient stability, contrast allergies, kidney function, and time delays, limit its utilization. We report a case of AAO in which point-of-care ultrasound (POCUS) expedited definitive diagnosis and treatment. We will review the current literature regarding the role of POCUS in the diagnosis of aortic pathology as well.

## CASE REPORT

An 86-year-old female with a past medical history significant for hypertension, congestive heart failure, aortic and mitral valve regurgitation status post repair, transient ischemic attack without residual neurologic deficits, and atrial fibrillation not on anticoagulation presented to an urban, academic emergency department (ED) with acute onset bilateral lower extremity pain, weakness, and paresthesia. The symptoms began two hours prior to arrival. The patient denied abdominal pain, back pain, fever, and recent trauma.

The patient arrived in moderate distress with an irregularly, irregular pulse of 65 beats per minute and a blood pressure of 187/109 millimeters of mercury. Other vital signs and the patient’s blood sugar were within normal limits. During initial triage, physical examination revealed new onset right (4/5) and left (2/5) lower extremity weakness. Neurological exam was otherwise unremarkable. A stroke alert was initiated immediately. CT head revealed no acute pathology. Upon return to the ED, the treating physician noted cool extremities with absent bilateral dorsalis pedis and posterior tibial pulses. Abdominal exam was benign. No rash or signs of trauma were evident. We then considered alternative etiologies of our patient’s bilaterally lower extremity weakness, including aortic pathologies.

**Image 1:**
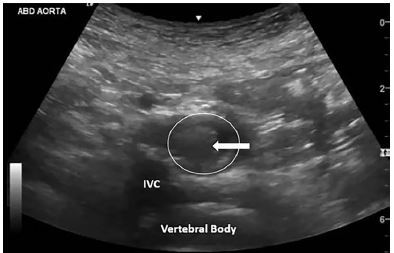
Point-of-care ultrasound transverse image of distal aorta (white circle) with hyperechoic intraluminal content (white arrow), representing thrombus.

**Image 2:**
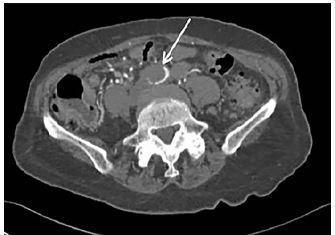
Computed tomography angiography axial image demonstrating occlusive thrombus (white arrow) in the distal abdominal aorta just proximal to the aortic bifurcation.

POCUS of the abdominal aorta demonstrated an occlusive intraluminal echogenicity originating just proximal to the iliac bifurcation (Image 1 and Video). Vascular surgery was consulted immediately for AAO. Emergent CT angiography of the inferior abdomen and bilateral lower extremities confirmed the aortoiliac occlusive thrombus (Image 2). The soft tissues demonstrated no evidence of myonecrosis. Laboratory values were unremarkable.

The patient was taken directly to the operating room where she underwent successful thrombectomy of the aorta, iliac, and femoral arteries. A transesophageal echocardiography did not demonstrate left atrial or ventricular thrombus. The patient had an unremarkable hospital course. Upon discharge to a skilled nursing facility, distal lower extremity pulses were present on Doppler exam, and the patient was ambulatory with mild residual bilateral lower extremity weakness.

## DICUSSION

Chronic aortic occlusive disease is a consequence of atherosclerotic disease, and has a reported incidence of 1–8%.^2^ The aortoiliac vessels are among the most common sites of chronic atherosclerosis, and risk factors for aortoiliac thrombotic disease are similar to those of other peripheral artery disease, including smoking, hyperlipidemia, hypertension, and diabetes.^7^ Chronic disease often presents with intermittent signs and symptoms of claudication.

Unlike chronic occlusive disease, our patient presented with acute onset lower extremity weakness and pain, consistent with AAO. This is a separate entity from chronic aortic occlusive disease, and has a reported mortality ranging between 21–74%.^2^–^5^ Etiologies include thrombosis (51.5%–91.6%), embolism, or aneurysmal disease.^2^–^5^ The patient did not have an aortic aneurysm or a history of claudication; however, she may have had pre-existing aortoiliac thrombosis. Given the patient’s medical history and lack of anticoagulation, the acute occlusion was likely secondary to embolic disease. Furthermore, the lower extremity motor deficits were subsequent to acute anterior spinal artery syndrome due to occlusion of the radicular artery of Adamkiewicz, which originates between the ninth thoracic vertebrae (T9) to T12 to supply the spinal cord, which was the level of the patient’s occlusion.^7^

The initial presentation of lower extremity deficits can mislead providers. Several case reports describe the mischaracterization of chronic aortic occlusive disease as sciatica, and acute aortic occlusive disease as a cerebrovascular accident or spinal cord myelopathy.^8^–^10^ Diagnostic uncertainty delays revascularization and increases morbidity and mortality as ischemic complications propagate, including neurologic deficits, amputation, renal failure, and mesenteric ischemia.^5^ In a case series of AAOs, Meagher et al. reported a mean delay of 24 hours from presentation to diagnosis.^9^ Dossa et al. reported 11% of patients had either a neurologic or neurosurgical consultation prior to the final diagnosis of AAO.^4^ Thorough physical examination and early use of POCUS facilitates recognition of vascular occlusion, redirecting diagnostic momentum away from neurogenic etiologies. It is noteworthy that the majority of AAOs occur infrarenal (75.8%–94.8%).^2^–^3^ The clinician is advised to extend the scan distal to the bifurcation of the aorta to ensure complete visualization of the entire abdominal aorta. Typically, thrombus is best visualized along the anterolateral wall of the abdominal aorta. The use of color flow and pulsed-wave Doppler to aid visualization and to differentiate between the aorta and inferior vena cava is also recommended.

**CPC-EM Capsule**

What do we already know about this clinical entity?

Acute aortic occlusion is a rare condition with high morbidity and mortality, varied presentations, and is often challenging to diagnose.

What makes this presentation of disease reportable?

The use of point-of-care ultrasound (POCUS) made the rapid diagnosis of acute aortic pathology masquerading as a neurologic presentation.

What is the major learning point?

Consider aortic pathology in patients with sudden onset lower extremity weakness; POCUS can be used to evaluate for abdominal aortic thrombus.

How might this improve emergency medicine practice?

POCUS can expedite diagnosis of acute aortic pathology and decrease time to definitive management.

Emergency physicians have been using POCUS for the diagnosis of aortic pathology for over 20 years. In fact, the 2016 American College of Emergency Physicians policy statement, Ultrasound Guidelines: Emergency, Point-of-care, and Clinical Ultrasound Guidelines in Medicine, mandates the sonographic evaluation of the abdominal aorta as a core emergency medicine (EM) competency.^11^ Numerous studies have validated sensitivities from 94–100% for the detection of abdominal aortic aneurysms with negative predictive values between 98.6%–100%.^12^–^15^ Costantino et al. demonstrated that the accuracy of EM resident-performed ultrasound is within 4.4 millimeters of CT evaluation of abdominal aortic aneurysms.^12^ More recently, Gibbons et al. established a POCUS aortic dissection protocol with 100% and 93.7% sensitivities for the detection of Stanford type A and Stanford type B aortic dissections, respectively.^16^ Furthermore, Pare et al. reported a mean reduction of 146 minutes in time to diagnosis of Stanford type A aortic dissections when implementing a POCUS-first approach.^17^

Although no formal studies exist evaluating the diagnostic accuracy of POCUS at identifying aortic occlusions, this case report illustrates a further extension of beside ultrasound in evaluating emergent aortic pathology.

## CONCLUSION

Acute aortic occlusion is a rare but potentially devastating vascular emergency. Emergency physicians should consider this aortic pathology in patients presenting to the ED with acute onset lower extremity neurovascular deficits. Diagnostic delay impedes time to revascularization and portends worse patient outcomes with morbidity and mortality rates between 21–74%.^2^–^5^ POCUS is a rapid, accurate, and non-invasive diagnostic imaging modality for patients presenting with aortic pathology. Given its high sensitivity for identifying aneurysms, dissections, and intraluminal thrombus, POCUS is the ideal screening exam for emergent aortic pathology.^12^–^17^

## Supplementary Information

Video.
